# Thermodynamics and Kinetics of Supramolecular Complex Formation between Carboxymethylcellulose and Lactoferrin

**DOI:** 10.1021/acsomega.6c02697

**Published:** 2026-07-07

**Authors:** Yara Luiza Coelho, Isabela A. Marques, Álvaro Javier Patiño-Agudelo, Hauster Maximiler C. de Paula, Eliara A. Hudson, Ana Clarissa S. Pires, Luis Henrique M. da Silva

**Affiliations:** † Advanced Thermokinetics of Molecular Systems (ATOMS) Group, Chemistry Department, Federal University of Viçosa, PH Rolfs Avenue, 36570-000 Viçosa-MG, Brazil; ‡ Department of Food Technology, Federal University of Viçosa, Av. P. H. Rolfs s/s, 36570900 Viçosa-MG, Brazil

## Abstract

In this study, the kinetics and thermodynamics of the interaction between bovine lactoferrin (BLF) and sodium carboxymethylcellulose (CMC) were investigated using surface plasmon resonance (SPR) and steady-state fluorescence spectroscopy (FS). SPR measurements suggested that the formation of the BLF-CMC complex occurs without significant conformational changes in the biopolymers (*k*_a_ ∼ 10^5^ L mol^–1^ s^–1^ and *k*_d_ ∼ 10^–2^ s^–1^). In both techniques, the binding constant was on the order of ∼10^6^ mol^–1^ L and Δ*G* ^o^< 0. Although the SPR data indicated that BLF-CMC complex formation is entropically driven (57 ≤ *T*Δ*S*_SPR_^o^ ≤ 60 kJ mol^–1^), the FS assays showed exothermic Δ*H*_FS_^o^ values (−96 and −31 kJ mol^–1^) up to 20 °C and endothermic (39, 111, and 187 kJ mol^–1^) at temperatures ≥ 25 °C, while the *T*Δ*S*_FS_^o^ values ranged from −58 to 230 kJ mol^–1^. This study provides useful insights into the formation of soluble BLF-CMC complexes, which may facilitate their application in the food industry.

## Graphical abstract

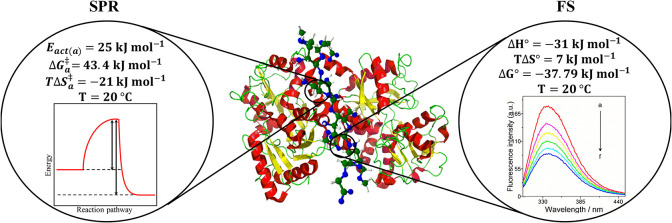

## 1 Introduction

Protein–polysaccharide interactions are fundamental physicochemical phenomena underlying numerous biological processes, including protein transcription, antigen–antibody recognition, and enzymatic channeling.^1–5^ In addition, the complexation of supramolecular structures containing these biomolecules plays a crucial role in various industrial applications, such as encapsulation processes,^6^ the design of multilayered systems,^7^ the formation and stabilization of food emulsions,^8^ the development of novel food gels,^9^ and the recovery of proteins from industrial byproducts.^10^ Therefore, a proper understanding of protein–polysaccharide interactions is essential for the design and development of new functional systems and has become a subject of continuous investigation.^11^

Depending on the charge density, molecular weight, and chemical nature of the biopolymers, as well as the stoichiometric ratio between the macromolecules and the experimental conditions (e.g., pH and ionic strength), the association between proteins and polysaccharides may lead to the formation of either soluble complexes or insoluble microaggregates (coacervates or precipitates).^12–15^ The formation of soluble protein–polysaccharide complexes offers several potentially valuable applications.^16^ Accordingly, in recent years, lactoferrin-polysaccharide complexation has attracted considerable attention, primarily due to the micro- and nanoencapsulation capabilities of these materials.^17^

Lactoferrin is an iron-binding glycoprotein present in various mammalian biological fluids and plays an important role in host defense due to its antioxidant, antimicrobial, and anti-inflammatory properties. Human and bovine lactoferrin share a similar structure and consist of 691 and 681 amino acid residues, respectively, of which 10 and 13 are tryptophan (Trp) residues. The three-dimensional structure comprises two lobes (N and C), each subdivided into two domains (N1, N2, C1, and C2), forming a cleft where a ferric ion (Fe^3+^) binds strongly (*K*_b_ ∼ 10^20^).^18^ In addition to these structural features, lactoferrin exhibits a relatively high isoelectric point (pH 8–9), indicating that it carries a net positive charge over a wide pH range.^19,20^ Consequently, bovine lactoferrin (BLF) can form soluble complexes with negatively charged polysaccharides, such as carboxymethylcellulose (CMC).

CMC is a cellulose-derived polysaccharide composed of linear β-(1→4)-linked *D*-glucopyranose units.^21–25^ The presence of hydroxyl and carboxylate groups confers a negative charge to CMC, enabling its interaction with proteins to form complexes exhibiting physicochemical and functional properties distinct from those of the individual macromolecules. Both CMC and BLF are environmentally friendly, nontoxic, and abundant biopolymers, which accounts for their widespread use in various technological applications.^26,27^ Complex or conjugate formation can enhance the stability of BLF under harsh conditions, such as elevated temperatures, high ionic strength, and extreme pH.^28^ Furthermore, these systems may be employed in the design of colloidal delivery platforms for the encapsulation, protection, and controlled release of bioactive compounds.^29^

Owing to the biological significance of BLF-polysaccharide interactions and the industrial potential of such systems, numerous studies have been reported in the literature. However, a critical analysis of both original research and review papers reveals that some fundamental aspects, particularly the kinetics and thermodynamics of the interactions between these biopolymers, remain to be fully elucidated.^30–33^

In this work, the kinetics and thermodynamics of the interaction between these two important biopolymers were investigated using steady-state fluorescence spectroscopy (FS) and surface plasmon resonance (SPR) to gain deeper insight into the formation mechanism of BLF-CMC soluble complexes. This study provides a comprehensive understanding of the molecular interactions between BLF and CMC, as well as valuable information for the rational design and development of new supramolecular systems with strategic applications across various scientific and technological fields. Unlike sulfated polysaccharides such as carrageenan or more rigid alginate structures, CMC is a flexible cellulose-derived polyelectrolyte whose charge density and hydration behavior are strongly influenced by the degree of substitution. These characteristics can substantially affect solvent organization, chain adaptability, and intermolecular binding thermodynamics during protein complexation. To our knowledge, few studies have comparatively investigated both global binding thermodynamics and local fluorophore-centered interactions for BLF–polysaccharide systems using complementary SPR and FS approaches.

## 2 Materials and Methods

### 2.1 Materials

Sodium carboxymethyl cellulose (CMC, Sigma-Aldrich, product 419281) with average molecular weight of ∼250 kDa and degree of substitution (DS) of 1.2 was used in all experiments. Considering the average molar mass of the substituted repeating units, the polymer chains are estimated to contain on the order of 10^3^ repeating units. CMC stock and working solution concentrations were calculated and expressed in terms of repeating monomeric units; thus, the molar concentration of CMC was obtained from the mass concentration divided by the average molar mass of the substituted repeating unit (∼258 g mol^–1^ for DS = 1.2), providing an effective concentration of accessible carboxymethylated segments in solution. Bovine milk lactoferrin (BLF), sodium acetate, and acetic acid were purchased from Sigma-Aldrich (St. Louis, MO). Research-grade CM5 sensor chips and the coupling reagents (N-ethyl-N′,N′-dimethylaminopropylcarbodiimide (EDC), *N*-hydroxysuccinimide (NHS), and ethanolamine hydrochloride (1 mol L^–1^, pH 8.5) were purchased from GE Healthcare (Pittsburgh, PA).

### 2.2 BLF-CMC Interactions Investigated by Steady-State Fluorescence Spectroscopy (FS)

All the fluorescence spectra were measured using a Cary Eclipse Fluorescence Spectrophotometer (Agilent Technologies, Santa Clara) equipped with a thermostat bath and 1 cm path-length quartz cuvettes. The temperature was controlled at 15, 20, 25, 30, and 35 °C. The Trp residues of BLF were excited at 295 nm using excitation and emission slit widths of 2.5 and 5.0 nm, respectively, and a photomultiplier tube (PMT) voltage of 750 V. Emission spectra were collected over the range 302–500 nm at a scan rate of 600 nm min^–1^. Measurements were performed at pH 4.0, using a fixed BLF concentration (1.97 × 10^–5^ mol L^–1^) in the presence of CMC at concentrations ranging from 0 to 1.7 × 10^–4^ mol L^–1^. pH 4.0 was selected because under these conditions BLF remains highly protonated whereas CMC preserves negatively charged carboxymethyl groups, favoring detectable protein–polysaccharide complex formation under controlled electrostatic conditions.

Since CMC exhibits absorption at wavelengths close to the excitation and emission wavelengths used in this study, all BLF fluorescence intensities measured in the presence of CMC were corrected according to eq 1 ^34^

1 $$F_{\mathrm{corr}}=F_{\mathrm{obs}}\cdot 10^{-A_{\mathrm{ex}}{-d}_{\mathrm{ex}}/2-A_{\mathrm{em}}{-d}_{\mathrm{em}}/2}$$

Where *F*_corr_ is the fluorescence intensity after correction for the inner-filter effect; *F*_obs_ is the fluorescence intensity measured by the spectrofluorometer; *A*_ex_ and *A*_em_ are the changes in absorbance at the excitation and emission wavelengths, respectively, caused by the addition of CMC (measured using a 1 cm path-length cuvette); and *d*_ex_ and *d*_em_ are optical path lengths in the excitation and emission directions (in cm), respectively.

### 2.3 BLF-CMC Interactions Investigated by Surface Plasmon Resonance (SPR)

The kinetic and thermodynamic parameters for the interactions between CMC and BLF were obtained by SPR using a Biacore X100 instrument (GE Healthcare, Pittsburgh, PA). Prior to the experiments, BLF was immobilized on a CM5 sensor chip using a Biacore amine coupling kit, according to the manufacturer’s instructions. Briefly, the CM5 chip surface was activated for 7 min with EDC/NHS, after which the remaining activated carboxyl groups were blocked with ethanolamine for an additional 7 min. BLF (15 μg mL^–1^) prepared in 0.01 mol L^–1^ sodium acetate buffer (pH 4) was then injected for immobilization. BLF was immobilized at relatively low level to minimize potential mass transport limitations and surface crowding effects. The immobilization level of ∼3864 RU corresponds to an estimated BLF surface density of ∼3.9 ng mm^–2^ (∼48 fmol mm^–2^), corresponding to an average intermolecular spacing on the order of tens of nanometers. These values suggest moderate surface coverage, helping to minimize excessive steric crowding while maintaining measurable SPR responses. In experiments with immobilized BLF, one flow cell was used as a reference surface; this surface was prepared as described above, but without BLF immobilization.

It should be noted that immobilization of BLF by EDC/NHS amine coupling may partially modify the surface distribution of positively charged amino groups because lysine ε-amines involved in covalent attachment can also participate in electrostatic interactions with CMC. Consequently, the immobilized protein surface may not fully reproduce the electrostatic environment of BLF in solution. Although BLF retains multiple accessible cationic regions after immobilization, the SPR-derived kinetic and thermodynamic parameters may partially reflect effects associated with immobilization-induced surface heterogeneity.

BLF-CMC interaction experiments were carried out at pH 4.0 and at temperatures ranging from 12 to 28 °C. CMC solutions in the concentration range 3.0 × 10^–6^ to 3.4 × 10^–6^ mol L^–1^ were prepared in pH 4.0. For each binding experiment, CMC solutions at defined concentrations were injected over both the sample (immobilized BLF) and reference (without BLF) flow cells to correct for bulk refractive index changes, systematic noise, and instrument drift. Prior to each CMC binding cycle, buffer at pH 4.0 was injected to establish a stable baseline.

## 3 Results and Discussion

### 3.1 Surface Plasmon Resonance (SPR)

#### 3.1.1 Kinetics of BLF-CMC Stable Complex Formation

In general, protein–polysaccharide (P–P) interactions yield insoluble molecular aggregates, such as coacervates and conjugates, depending on thermodynamic conditions including pH, concentration, and ionic strength.^13,29,35^ However, stable and soluble P–P complexes can be obtained by modulating these thermodynamic conditions, thereby enabling the determination of the kinetic and thermodynamic parameters governing complex formation. Thermodynamic binding between proteins and polysaccharides in solution has been investigated using different techniques, such as isothermal titration microcalorimetry and fluorescence spectroscopy (FS).^36^ However, kinetic data for these interactions remain scarce. SPR is a real-time technique that allows the examination of intermolecular interactions between biomolecules. It provides kinetic parameters for the association of free biomolecules and the dissociation of thermodynamically stable complexes through the analysis of sensorgram data, in which the resonance units (RU) are expressed as a function of time. Figure 1 illustrates the sensorgrams for BLF-CMC interaction at 25 °C and pH 4.0. Similar sensorgrams were obtained for the other five different temperatures (Figure S1).

**Figure 1:**
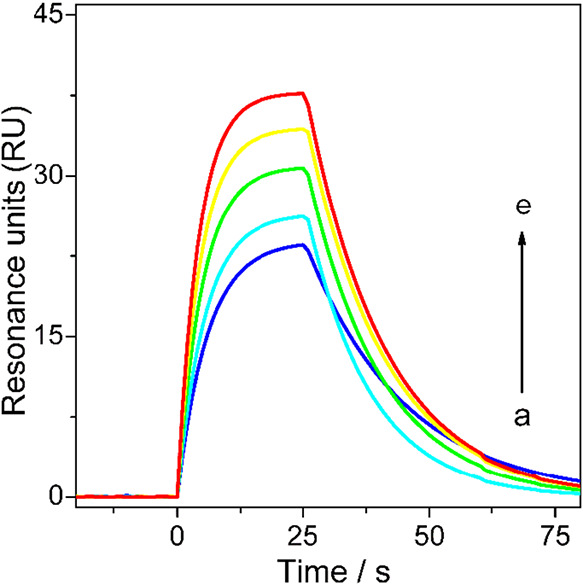
Sensorgrams for BLF interacting with CMC at different concentrations (a–e): 3.0 × 10^–6^–3.4 × 10^–6^ mol L^–1^), flowing over a CM5 low-density protein-immobilized sensor chip surface (3864 RU) at pH 4.0 and 25 °C.

The sensorgrams reveal changes in the RU values over time and can be related to the association between free BLF and CMC molecules to form the BLF-CMC complex, as well as to the dissociation of this complex. This process can be represented by the chemical equilibrium described in eq 2

2 BLF+CMC⇌BLF‐CMC

The kinetic parameters were obtained by globally fitting the RU versus time data (eqs 3 and 4) to a 1:1 binding model. Although CMC is a multivalent polydisperse polysaccharide capable of establishing multiple simultaneous interactions with BLF, the 1:1 Langmuir model was adopted as an effective phenomenological model to describe the dominant global binding behavior observed in the SPR sensorgrams. More complex interaction models were not employed because they did not significantly improve fitting quality or produced unstable correlated parameters under the experimental conditions.

3 $$\mathrm{RU}\left(t\right)=\mathrm{RU}\left(t_{m}\right)e^{-k_{d}\left(t-t_{m}\right)}$$

4 $$\mathrm{RU}\left(t\right)={\mathrm{RU}}_{\max}\left(t_{\infty }\right)\left[1-e^{-k_{\mathrm{obs}}\left(t\right)}\right]$$

Where *k*_d_ is the dissociation rate constant, *t*_m_ is the time at the beginning of the descending phase, i.e., when only the dissociation process occurs, *k*_obs_ is the observed rate constant, and [RU_max_ (*t*_*∞*_)] is the SPR response at BLF saturation by CMC. The *k*_obs_ value was used to determine the association rate constant (*k*_a_) from the slope of the *k*_obs_ versus [CMC] curve (Figure S2). The *k*_a_ and *k*_d_ values for the BLF-CMC complexes at pH 4.0 and different temperatures are presented in Table S1.

Considering that *k*_a_ represents the number of BLF-CMC complexes formed per second and *k*_d_ denotes the fraction of complexes that dissociate per second, the high *k*_a_ values (1.4 ± 0.1 × 10^5^ – 2.8 ± 0.1 × 10^5^ L mol^–1^ s^–1^) and low *k*_d_ values (7.7 ± 0.1 × 10^–2^ – 9.3 ± 0.3 × 10^–2^ s^–1^) indicate that complex formation between BLF and CMC occurs without significant conformational changes in the biopolymer chemical structure. In binding processes involving pronounced molecular rearrangements and conformation changes, smaller *k*_a_ and larger *k*_d_ values are typically observed.^37^ The relatively fast association kinetics and moderate dissociation behavior suggest that large-scale conformational rearrangements may not be strictly required prior to complex formation, although local structural adaptation during binding cannot be excluded. Thus, it is likely that CMC interacts with surface sites of BLF, inducing only minor changes in the protein and/or polysaccharide conformation.

The *k*_a_ values determined in our study were of the same order of magnitude as those reported for antigen/antibody complexes (mAb/IgG pAb) at 25 °C (1.13 × 10^5^ L mol^–1^ s^–1^),^38^ and 3.8 times higher than those reported for actin-aplyronine A complexes with tubulin heterodimers (4.78 × 10^4^ L mol^–1^ s^–1^).^39^ On the other hand, antigen/antibody complexes are formed through highly specific interactions; therefore, their *k*_d_ values are significantly lower (2.23 × 10^–4^ s^–1^). In contrast, for heterodimer formation the *k*_d_ values are of the same order of magnitude (8.82 × 10^–2^ s^–1^) as those obtained in the present study.

The study of the temperature dependence of *k*_a_ and *k*_d_ provided important energetic parameters related to the formation of an activated BLF-CMC complex from the association of free BLF and CMC, and to the dissociation of the thermodynamically stable BLF-CMC complex, respectively. The plots of ln *k*_a_ or ln *k*_d_ versus 1/T (Figure S3) allowed the determination of the activation energies (*E*_act(*x*)_) for the association (*x* = a) and dissociation (*x* = *d*) processes (eq 5).

5 $$E_{\mathrm{act}\left(x\right)}\left(T\right)=-R\left(\frac{d\ln k_{x}}{dT}\right)$$

Where *k*_*x*_ is the association or dissociation rate constant (L mol^–1^ s^–1^ or s^–1^, respectively), *E*_act(*x*)_ is the activation energy (kJ mol^–1^), and *R* is the universal gas constant. The Arrhenius plots displayed a second-order polynomial dependence of ln *k*_a_ and ln *k*_d_ with 1/*T*, suggesting a multisite and multistep induced-fit process.^40,41^ The *E*_act_ values at each temperature are listed in Table 1.

**Table 1:** Energetic Parameters for the Formation of BLF-CMC Activated Complexes from the Association of Free BLF and CMC Biomolecules (a) and the Dissociation of Thermodynamically Stable BLF-CMC Complexes (d), at pH 4.0

	association phase (*a*)				dissociation phase (*d*)			
*T* °C	*E*_act(a)_	Δ*H*_a_^‡^	Δ*G*_a_^‡^	*T*Δ*S*_a_^‡^	*E*_act(d)_	Δ*H*_d_^‡^	Δ*G*_d_^‡^	*T*Δ*S*_d_^‡^
	kJ mol^–1^							
12	–42 ± 1	–44 ± 1	42 ± 1	–86 ± 1	–70 ± 1	–73 ± 1	75.8 ± 0.1	–148 ± 1
16	–15 ± 1	–17 ± 1	43 ± 1	–60 ± 1	–38 ± 1	–40 ± 1	77.7 ± 0.1	–117 ± 1
20	25 ± 1	22 ± 1	43 ± 1	–21 ± 1	4 ± 1	1 ± 1	79.1 ± 0.1	–78 ± 1
24	78 ± 2	75 ± 2	43 ± 1	32 ± 2	56 ± 1	53 ± 1	79.7 ± 0.1	–27 ± 1
25	93 ± 2	90 ± 2	43 ± 1	47 ± 2	70 ± 2	68 ± 2	79.8 ± 0.1	–12 ± 2
28	142 ± 2	140 ± 2	42 ± 2	98 ± 3	116 ± 2	114 ± 2	79.7 ± 0.1	34 ± 2

The *E*_act(a)_ and *E*_act(d)_ values increased exponentially with temperature (Figure S4). At all studied temperatures, the *E*_act(a)_ values were higher than the *E*_act(d)_ values, indicating that formation of the activated complex from the free BLF and CMC molecules requires more energy to overcome the potential energy barrier than dissociation process. The negative *E*_act(a)_ and *E*_act(d)_ values observed at low temperatures can be explained by considering that *E*_act(*x*)_ (*x* = a or d) can be divided into three submolecular processes, as indicated in eq 6

6 $$E_{\mathrm{act}\left(x\right)}=E_{\mathrm{act},\mathrm{des}}+E_{\mathrm{act},\mathrm{int}}+E_{\mathrm{act},\mathrm{conf}}$$

where *E*_act,des_ is the energy absorbed during the transfer of water molecules from the solvation shell of the ligand (protein or polysaccharide) to the bulk solution, *E*_act,conf_ is the energy absorbed during biopolymer conformational changes induced by the interactions, and *E*_act,int_ is the energy released due to protein–polysaccharide interactions.

At temperatures < 16 °C, *E*_act,des_ and *E*_act,conf_ are lower than the magnitude of *E*_act,int_. In this temperature range, the average molecular kinetic energy (<*E*_k_>) is insufficient to significantly alter the hydrogen-bonding network of water molecules; therefore, the three-dimensional water structure in the biomolecule solvation shell remains similar to that in the bulk solution. In addition, the rotational potential barrier within the biopolymer structure is high enough to prevent conformational changes induced by molecular collisions or intermolecular BLF-CMC interactions. Consequently, *E*_act(*x*)_< 0, as it is dominated by the energy released from specific protein–polysaccharide interactions. However, with increasing temperature, *E*_act(*x*)_ becomes positive because the energy required to disrupt the biopolymer solvation shell and to induce macromolecular conformational changes contributes significantly to *E*_act(*x*)_. Moreover, at higher temperatures, the energetic and structural differences between water molecules in the solvation shell and those in the bulk solution become more pronounced. Additionally, < *E*_k_> becomes sufficiently high to overcome the rotational potential energy barrier at the interacting sites of the biopolymers through energy transfer by molecular collisions. It should be noted that, due to the polymeric nature of CMC and the possibility of multisite interactions, the calculated activation parameters represent apparent values associated with the overall interaction process rather than elementary activation energies for an idealized 1:1 binding mechanism.

We investigated the relative contributions of intermolecular interactions and configurational changes in the system to the formation of the BLF-CMC activated complex during the association and dissociation phases. Thus, the Gibbs free energy change (Δ*G*_*x*_^‡^), enthalpy change (Δ*H*_*x*_^‡^), and entropy change (Δ*S*_*x*_^‡^) of activation for both phases were determined from eqs 7, 8, and 9, respectively (Table 1).

7 $$\Delta G_{x}^{‡}=-\mathrm{RT}\ln\left(\frac{k_{x}h}{K_{B}T}\right)$$

8 $$\Delta H_{x}^{‡}=E_{\mathrm{act}\left(x\right)}-\mathrm{RT}$$

9 $$T{\Delta S}_{x}^{‡}={\Delta H}_{x}^{‡}-{\Delta G}_{x}^{‡}$$

where *K*_B_ and *h* are the Boltzmann’s and Planck’s constants, respectively.

The Δ*G*_*x*_^‡^ values were positive for both the association and dissociation phases at all temperatures. However, in contrast to the temperature dependence observed for *E*_act(*x*)_, the Δ*G*_*x*_^‡^ remained nearly constant with temperature. In general, when the activation Gibbs free energy change for protein–ligand binding processes is weakly dependent on temperature, isokinetic compensation (IKC) is assumed. In this phenomenon, large changes in the activation entropy term are accompanied by proportional changes in the activation enthalpy component, resulting in only small variations in the overall free energy. Figure 2 illustrates the linear relationship between the Δ*H*_*x*_^‡^ and *T*Δ*S*_*x*_^‡^ values obtained in this study, with slopes of 1.021 ± 0.008 for the association (*x* = a) and 1.006 ± 0.005 for the dissociation (*x* = d) processes.

**Figure 2:**
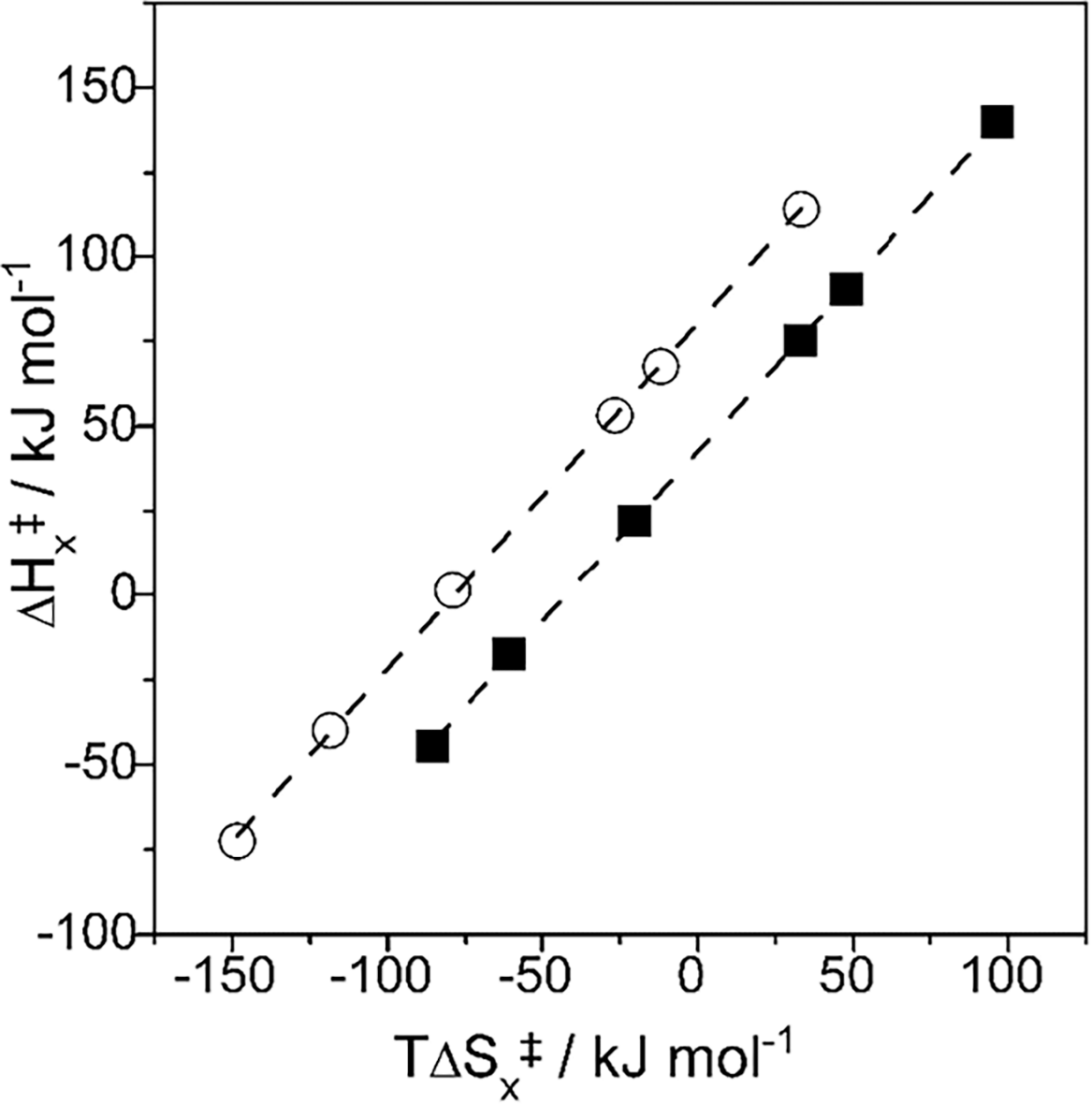
Isokinetic compensation for the BLF-CMC binding process during the association (■) and dissociation (○) phases.

The observed IKC for BLF-CMC binding supports the molecular explanation for the temperature dependence of *E*_act(*x*)_. The interaction between free BLF and CMC biomolecules leads to solvent reorganization, caused by the disruption of water molecules solvating the macromolecules and the formation of water–water interactions in the bulk solution.^42^ This solvent reorganization promotes a proportional increase in both the activation enthalpy and entropy of the system. Another source of IKC arises from conformational restriction of BLF and CMC in the bound state, which reduces the distribution of accessible conformational energies in the BLF-CMC complex and consequently decreases conformational entropy. As a result, the decrease in activation entropy becomes proportional to the reduction in activation enthalpy upon binding. This molecular explanation for the IKC contribution can be applied to both the association of free BLF and CMC biomolecules and the dissociation of the thermodynamically stable BLF-CMC complex to form the activated complex.

#### 3.1.2 Thermodynamics of BLF-CMC Complex Formation

The thermodynamic parameters of complex formation, such as the standard Gibbs free energy change (Δ*G* ^o^), standard enthalpy change (Δ*H* ^o^), and standard entropy change (Δ*S* ^o^), are useful to understanding the main forces involved in the complex formation process between proteins and different molecules.^43^ Therefore, these parameters were determined to provide comprehensive insight into the stability and the driving forces of the BLF-CMC complex. To calculate the binding constant (*K*_b_^SPR^) for the stable BLF-CMC complex from the SPR kinetic data, we used the well-established relationship *K*_b_^SPR^ = *k*_a_/*k*_d_ at each temperature. Subsequently, the Δ*H* ^o^ values were obtained using the van’t Hoff approach (ln *K*_*b*_^*SPR*^ versus 1/T; Figure S5 and eq 10).


10 $$\mathrm{In}\left(\frac{K_{b}\left(T_{2}\right)}{K_{b}\left(T_{1}\right)}\right)=-\frac{\Delta H^{{}^{\circ}}}{R}(\frac{1}{T_{2}}-\frac{1}{T_{1}})$$

The Δ*G* ^o^ and *T*Δ*S* ^o^ values were calculated from eqs 11 and 12, respectively. The thermodynamic parameters of BLF-CMC complex formation determined by SPR at pH 4.0 are presented in Table 2.

11 $${\Delta G}^{0}=-\mathrm{RT}\ln K_{b}$$

12 $$T\Delta S^{{}^{\circ}}={\Delta H}^{0}-{\Delta G}^{0}$$

**Table 2:** Thermodynamic Parameters for the Formation of BLF-CMC Complexes at Different Temperatures and pH 4.0, Obtained from Surface Plasmon Resonance (SPR) Experiments

	*K*_b_^SPR^	Δ*H*_SPR_^o^	Δ*G*_SPR_^o^	*T*Δ*S*_SPR_^o^
*T* °C	10^6^ mol^–1^ L		kJ mol^–1^	
12	1.8 ± 0.2	23 ± 1	–34.1 ± 0.2	57 ± 1
16	2.1 ± 0.1		–35.1 ± 0.1	58 ± 1
20	2.3 ± 0.2		–35.7 ± 0.2	59 ± 1
24	2.7 ± 0.2		–36.6 ± 0.2	60 ± 1
25	2.8 ± 0.1		–37.0 ± 0.1	60 ± 1
28	3.0 ± 0.2		–37.4 ± 0.1	60 ± 1

The *K*_b_^SPR^ values were on the order of 10^6^ mol^–1^ L and increased with increasing temperature. This demonstrated that the equilibrium BLF + CMC ⇌ BLF-CMC favored complex formation and that BLF-CMC complex formation occurred via energy absorption. The Δ*G*_SPR_^o^ values were negative and became more negative with increasing temperature, confirming that the BLF-CMC complexes are more stable at higher temperatures.

To determine the enthalpic and entropic contributions for the negative Δ*G*_SPR_^o^, Δ*H*_SPR_^o^ and *T*Δ*S*_SPR_^o^ values were also calculated (Table 2). The positive Δ*H*_SPR_^o^ and *T*Δ*S*_SPR_^o^ values indicate that the thermodynamic process of BLF-CMC complex formation is entropically driven and enthalpically unfavorable. This supports the hypothesis that the release of water molecules and counterions from the solvation shell of both biomolecules into the bulk solution plays an important role in complex formation. Although electrostatic interactions clearly contribute to BLF–CMC binding, the thermodynamic parameters suggest that additional nonelectrostatic contributions, including hydrogen bonding, desolvation effects, solvent reorganization, and local conformational rearrangements, may also play important roles in the complexation process. In addition, the positive entropy changes observed may be partially associated with counterion release and solvent reorganization accompanying electrostatic interactions between the oppositely charged biopolymers. To understand the relatively small contribution of the electrostatic interactions between the positively charged BLF and negatively charged CMC to the Δ*H*_SPR_^o^ value, since electrostatic interactions would be expected to produce negative Δ*H*_SPR_^o^ values, it is important to note that this parameter encompasses four molecular processes (eq 13)­

13 $${\Delta H}^{0}={\Delta H}_{\mathrm{desol}}^{0}+{\Delta H}_{\mathrm{BLF}-\mathrm{CMC}}^{0}+{\Delta H}_{H_{2}O-H_{2}O}^{0}+{\Delta H}_{\mathrm{conf}}^{0}$$

where Δ*H*_desol_^0^ is the positive enthalpic change associated with the disruption of water–water interactions in the solvation shells of the BLF and CMC biomolecules; Δ*H*_BLF–CMC_^0^ is the negative enthalpic change arising from the interaction between BLF and CMC; Δ*H*_H_2_O–H_2_O_^0^ is the negative enthalpic change due to the interactions between the water molecules in the bulk solution; and Δ*H*_conf_^0^ is the positive enthalpic change associated with conformational changes of the protein and CMC upon complex formation. The positive Δ*H*_SPR_^o^ values suggest that more energy is required to desolvate the BLF and CMC molecules and induce conformation changes in the ligand than the energy released from BLF-CMC interactions in the bulk solution.

### 3.2 Characterization of BLF–CMC Complex Formation by Fluorescence Spectroscopy

Although SPR is a highly sensitive technique for determining kinetic and thermodynamic binding parameters, it is limited to the analysis of proteins immobilized on a solid substrate. In contrast, the thermodynamic binding parameters of biomolecules interacting in solution can be obtained by FS. BLF contains 13 Trp residues that emit fluorescence when excited at 295 nm. However, interactions between proteins and other molecules, such as polysaccharides, in the vicinity of these residues (∼5 Å) often quench the intrinsic fluorescence of the protein,^44^ thereby enabling the use of FS for investigating interactions between BLF and various ligands. The fluorescence spectra of BLF in the presence of increasing concentrations of CMC at 25 °C and pH 4.0 are shown in Figure 3.

**Figure 3:**
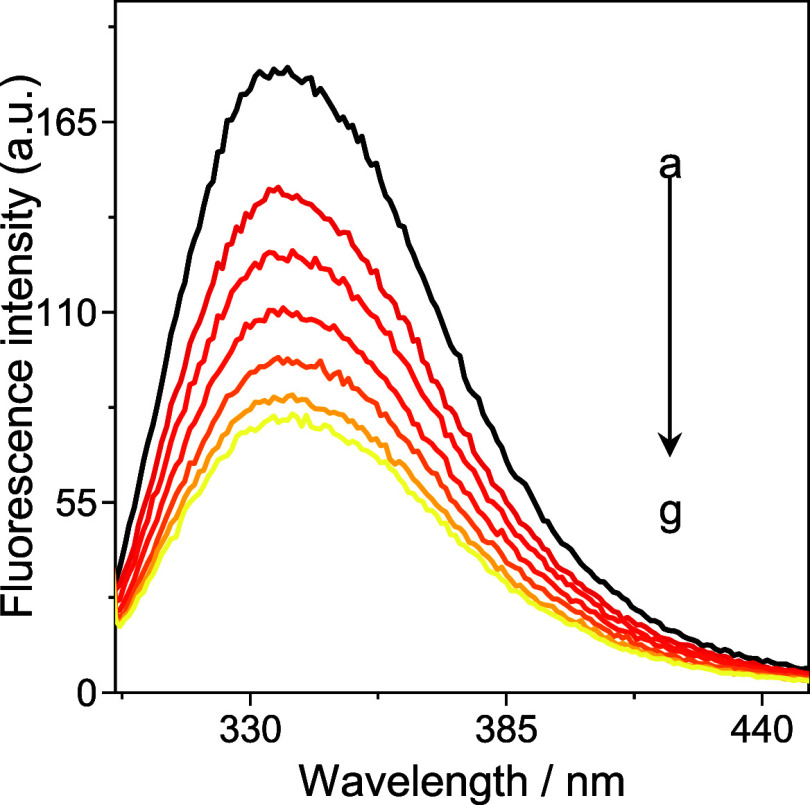
Fluorescence emission spectra of bovine lactoferrin (BLF, 1.97 × 10^–5^ mol L^–1^) in the absence and presence of increasing concentrations of sodium carboxymethylcellulose (CMC, (a) 0– (g)­1.7 × 10^–4^ mol L^–1^) at pH 4.0 and 25 °C. The spectra are shown from dark red to yellow according to increasing CMC concentration. The progressive changes in fluorescence intensity indicate the occurrence of interactions between BLF and CMC.

BLF fluorescence quenching increased with increasing CMC concentration, without displacement of the maximum fluorescence emission wavelength (λ_max_). This decrease in the fluorescence intensity of the fluorophore can occur through different mechanisms, classified as dynamic or static quenching. Dynamic quenching refers to a process resulting from collisions between the quencher and the fluorophore during the excited state, whereas static quenching refers to the formation of fluorophore-quencher complex in the ground state.^45^ To determine the mechanism responsible for BLF fluorescence quenching by CMC, the Stern–Volmer eq (eq 14)^46^ was used

14 $$\frac{F_{0}}{F}=1+k_{q}\tau_{0}\left[\mathrm{CMC}\right]$$

where *F* and *F*_o_ are the fluorescence intensities of BLF in the presence and absence of CMC, respectively; [CMC] is the concentration of CMC; *k*_q_ is the bimolecular quenching rate constant of the protein fluorescence; and τ_0_ (1.06 × 10^–9^ s) is the lifetime of the fluorophore (in this case, the excited Trp residues in BLF) in the absence of CMC.^47^ The *k*_q_ values were obtained from the plot of *F*_o_/ *F versus* [CMC] (Figure S6). The *k*_q_ values ranged from 6.8 ± 0.1 × 10^12^ to 11.4 ± 0.6 × 10^12^ L mol^–1^ s^–1^ (Table S2), which are much higher than the maximum rate constant for dynamic quenching (2 × 10^10^ L mol^–1^ s^–1^). This indicated that the quenching of BLF fluorescence is induced by complex formation between BLF and CMC.^48^ Assuming static quenching and that the binding sites in BLF are similar and independent, the binding constant (*K*_b_^*FS*^) and the stoichiometry of complex formation (*n*) can be determined using the double-logarithm regression model (eq 15).^49^

15 $$\log\frac{F_{0-}F}{F}=\log K_{b}^{\mathrm{FS}}+n\;\log\left[\mathrm{CMC}\right]$$

where *F*_o_ and *F* are the fluorescence intensities of BLF in the absence and presence of CMC, respectively; *n* is the stoichiometry number; *K*_b_^FS^ is the binding constant; and [CMC] is the CMC concentration. The *n* and *K*_b_^FS^ values were obtained from the slope and intercept, respectively, of the plot of *log F*_o_ – *F*/*F versus log* [CMC] (Figure S7). The *K*_b_^FS^and *n* values are listed in Table S2.

The *K*_b_^FS^ values (8.40 ± 0.01 × 10^6^–23.28 ± 0.01 × 10^6^ L mol^–1^) were of the same order of magnitude as those obtained by SPR (10^6^ L mol^–1^). For the FS experiments, the average stoichiometry number was 1.77, indicating that, on average, approximately 1.8 CMC monomeric units contribute to the local microenvironment surrounding the accessible Trp residues responsible for fluorescence quenching. This result is reasonable because the calculations of *K*_b_ and *n* were performed considering the concentration of monomeric units present in the CMC macromolecule. Notably, the average *n* value obtained by FS differs from that obtained by SPR, because two techniques probe different structural levels of interaction. In the SPR assays, *n* refers to the number of CMC macromolecules bound to one BLF biomolecule, reflecting a global binding stoichiometry. In contrast, in the FS assays, *n* represents the average number of CMC monomeric units interacting with the surroundings of the Trp residues.

Since CMC is a macromolecule composed of multiple repeating units distributed along a flexible chain, the interaction of a single CMC macromolecule with BLF may expose several segments to the vicinity of one or more Trp residues. Therefore, although the numerical values obtained by FS and SPR are different, they are physically consistent because they describe distinct structural scales of the same interaction process.

The thermodynamic parameters of complex formation were also determined by fluorescence (Δ*H*_FS_^*o*^, Δ*G*_FS,_^*o*^ and *T*Δ*S*_FS_^*o*^) using the previously described eqs (eqs 10–12). The van’t Hoff plot is presented in Figure S8, while the standard thermodynamic parameters for BLF-CMC complex formation determined by fluorescence are listed in Table 3.

**Table 3:** Standard Enthalpy Change (Δ*H*_FS_^o^), Standard Gibbs Free Energy Change (Δ*G*_FS_^o^), and Standard Entropy Change (*T*Δ*S*_FS_^o^) Obtained by Fluorescence Spectroscopy for BLF-CMC Complex Formation at pH 4.0

	Δ*H*_FS_^o^	Δ*G*_FS_^o^	*T*Δ*S*_FS_^o^
*T* °C	kJ mol^–1^		
15	–96 ± 1	–38.18 ± 0.00	–58 ± 1
20	–31 ± 1	–37.79 ± 0.01	7 ± 1
25	39 ± 1	–38.37 ± 0.01	77 ± 1
30	111 ± 2	–40.39 ± 0.00	151 ± 2
35	187 ± 2	–43.44 ± 0.00	230 ± 2

The Δ*G*_FS_^o^ values were all negative, indicating that the BLF-CMC complex is stable at all the studied temperatures. The stability of the BLF-CMC complexes determined by FS was ∼5 kJ mol^–1^ greater than that determined by the SPR technique.

The Δ*H*_FS_^o^ values increased linearly with temperature (Figure S9) and were exothermic up to a temperature of 20 °C and endothermic at temperatures ≥ 25 °C. The inversion of Δ*H*° values observed in the fluorescence experiments suggests a temperature-dependent balance between favorable intermolecular interactions and unfavorable structural/desolvation contributions. At lower temperatures, electrostatic interactions and hydrogen bonding between BLF and CMC likely dominate the binding energetics, resulting in exothermic complex formation. However, as temperature increases, energetic penalties associated with disruption of hydration shells and local conformational rearrangements surrounding the Trp residues become increasingly important. Under these conditions, the enthalpic cost associated with desolvation and structural perturbation exceeds the favorable interaction enthalpy, leading to positive Δ*H* values. The relationship between Δ*H*_FS_^o^ and *T* (Figure S10) allows the determination of Δ*C*p_FS_^0^ $\left({\Delta Cp}_{FS}^{0}=\frac{\partial {\Delta H}_{FS}^{0}}{\partial T}\right)$ . For the BLF-CMC complex formation process, Δ*C*p_FS_^0^ = 14.1 ± 0.1 kJ mol^–1^ K^–1^. This positive Δ*C*p_FS_^0^ value suggests that, despite possible conformational changes in the interacting biopolymers caused by the disruption of intramolecular interactions, the formation of new intermolecular interactions between BLF and CMC contributes to an increase in the potential energy component of the system.^50^

In addition, the enthalpic differences observed between SPR and FS measurements may also be associated with the distinct experimental conditions employed by the two techniques. In SPR experiments, BLF is immobilized on a dextran matrix, which may impose local conformational constraints and alter the hydration/desolvation balance at the protein interface during complex formation. In contrast, fluorescence spectroscopy probes the interaction in the free state in solution, where the protein retains greater conformational flexibility. Therefore, differences in Δ*H*° values and even inversion of the enthalpic contribution with temperature may partially reflect differences in protein environment and solvation between the two experimental approaches.

The values of the entropic term obtained by FS were very different from those determined by the SPR experiments. While the *T*Δ*S*_FS_^o^ values increased linearly with temperature (−58 ± 1 to 230 ± 2 kJ mol^–1^) in the former technique, the *T*Δ*S*_SPR_^*o*^ values showed little variation (∼60 kJ mol^–1^). This difference between the *T*Δ*S* ^o^ values obtained by the two techniques arises because SPR considers all interacting sites of both the CMC and BLF biomolecules. In contrast, FS only probes the interactions occurring between CMC binding sites and the Trp residues of BLF.

As observed in other protein–ligand interaction processes,^51^ the fluorescence results in this study exhibited an enthalpy–entropy compensation phenomenon (Figure S11). Notably, at first glance, the single line in Figure S11 fits all the data very well (*r* ^2^ ≅ 0.999). A possible explanation for this phenomenon is that enthalpy–entropy compensation in protein–ligand binding may originate from the formation and disruption of weak noncovalent interactions. Several factors appear to influence this compensation behavior, including the structural and thermodynamic properties of the solvent (e.g., hydrophobic effects, solvation, desolvation, and local water structure), the flexibility of the binding sites or regions surrounding the localized site, the molecular structure of the ligand, and changes in intermolecular forces during the binding process.^52^

For instance, BLF-CMC complex formation resulting from favorable noncovalent interactions between the macromolecules may lead to a negative enthalpy change; however, this can be accompanied by a negative entropy change due to the restricted mobility of the interacting partners, resulting in only a small variation in Δ*G* ^o^. On the other hand, free ligand desolvation is usually accompanied by an entropy gain and an enthalpic penalty (i.e., a positive enthalpy change). In addition, the interaction of the ligand with BLF may induce conformational changes at the protein binding site. This can result in an entropy gain due to an increase in the conformational degrees freedom of the protein segments, along with a concomitant increase in Δ*H* ^o^ caused by the disruption of intramolecular interactions among the protein amino acid residues, so that Δ*G* ^o^ remains nearly constant.

## 4 Conclusions

The interaction between lactoferrin and CMC was investigated by SPR and FS techniques. The results revealed that CMC binds to BLF with high affinity (10^6^ mol^–1^ L), forming more stable complexes at higher temperatures. Intriguingly, although electrostatic interactions contributed to BLF-CMC binding, nonpolyelectrolytic interactions appear to play a dominant role in the binding process. SPR measurements provided kinetic parameters associated with the formation of a BLF-CMC activated complex through the association of free BLF and CMC or dissociation of stable BLF-CMC complexes. At temperatures ≤ 16 °C, BLF-CMC activated complex formation is mainly driven by the energy released from specific interactions between the protein and the polysaccharide. However, with increasing temperature, the energy required for the release of the biopolymer solvation shells, as well as for conformational changes, also contributes to the activation energy. Although the present work focused on a single pH condition to enable detailed thermodynamic and kinetic characterization, extension to broader pH ranges, especially near the isoelectric region of BLF, may provide additional insight into the balance between electrostatic, desolvation, and conformational contributions during complex formation. Overall, this work provides a detailed kinetic and thermodynamic characterization of BLF-CMC interactions, offering important insights into the behavior of protein–polysaccharide systems. The high affinity and temperature-dependent stability observed for the BLF-CMC complexes suggest that these systems may be relevant for the development of food formulations containing protein–polysaccharide assemblies, particularly under processing and storage conditions involving temperature variations. In this context, the kinetic and thermodynamic parameters obtained in this work may contribute to the rational design and optimization of such formulations.

## Supplementary Material



## Notes

Sensorgrams for BLF interacting with CMC (Figure S1); plot of as a function of CMC concentration (Figure S2); Arrhenius plot (Figure S3); activation energy versus temperature plot (Figure S4); the van’t Hoff plot (Figures S5 and S8); Stern–Volmer plots­(Figure S6); plot for the determination of and *n* values (Figure S7); versus T plot (Figures S9 and S10); enthalpy–entropy compensation (Figure S11); rate constants for the association (*k*_a_) of the free BLF and CMC biomolecules and the dissociation (*k*_d_) of the BLF-CMC stable complex at pH 4.0 and different temperatures (Table S1); bimolecular quenching rate constant (*k*_q_), binding constants (k_b_^FS^) and stoichiometry numbers (*n*) of complex formation between BLF and CMC at different temperatures and pH 4.0 (Table S2) (PDF)

## §

Colloid Chemistry Group, Institute of Chemistry, Federal University of Alfenas, Rua Gabriel Monteiro da Silva, 700, Alfenas, MG 37130–000, Brazil

## ∥

Department of Physical Chemistry, Institute of Chemistry, University of Campinas (UNICAMP), Campinas, SP 13083–970, Brazil.

## ⊥

Department of Chemistry, Federal University of Oeste do Pará, Santarém, PA 68040–070, Brazil.
