# Supplementary material for: Thermodynamics and Kinetics of Supramolecular Complex Formation between Carboxymethylcellulose and Lactoferrin
Source: ACS Omega. 2026 Jul 7;11(28):42188–96. doi: 10.1021/acsomega.6c02697 (PMC13393380; doi:10.1021/acsomega.6c02697)
Supplement: Supplementary file 1 [file ao6c02697_si_001.pdf]

Supplementary Material

Thermodynamics and kinetics of supramolecular complex formation between carboxymethylcellulose and lactoferrin

Yara Luiza Coelho[1,2], Isabela A. Marques[1], Álvaro Javier Patiño-Agudelo[1,3], Hauster Maximiler C. de Paula[1], Eliara A. Hudson[4], Ana Clarissa S. Pires[4], Luis Henrique M. da Silva[1*]

[1]*Advanced Thermokinetics of Molecular Systems (ATOMS) Group, Chemistry Department, Federal University of Viçosa, PH Rolfs Avenue, 36570-000 Viçosa-MG, Brazil.*

[2]*Colloid Chemistry Group, Institute of Chemistry Federal University of Alfenas, Gabriel Montero da Silva 700, 37130000, Alfenas-MG, Brazil*

[3]*Department of Physical Chemistry, Institute of Chemistry, University of Campinas, UNICAMP, Campinas, 13084-862, São Paulo, Brazil.*

[4]*Department of Food Technology, Federal University of Viçosa, Av. P. H. Rolfs s/s, 36570900, Viçosa-MG, Brazil.*

E-mails:
yara.coelho@unifal-mg.edu.br
marques.isabela100@gmail.com
patinoagudeloaj@gmail.com
hauster.campos@gmail.com
eliara.hudson@ufv.br
anaclarissasp@yahoo.com.br

**Corresponding author: luhen@ufv.br; Phone: +55 31 38993052; Fax: +55 31 38992175*

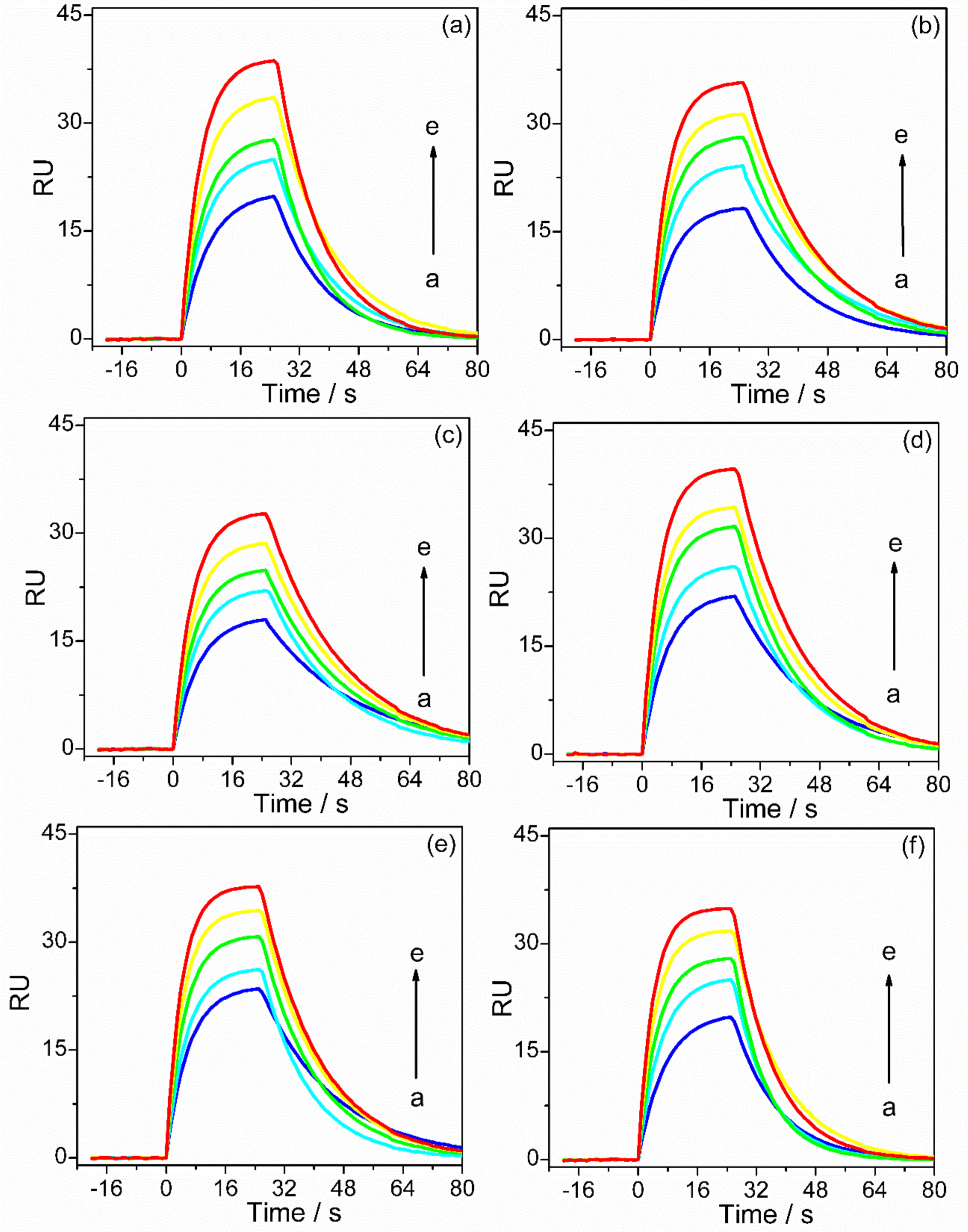

Figure S1. Sensorgrams for BLF interacting with CMC at different concentrations (3.0×10^{-6} – 3.4×10^{-6} mol L^{-1}) at pH 4.0 and different temperatures: (a) 12 °C, (b) 16 °C, (c) 20 °C, (d) 24 °C, (e) 25 °C, and (f) 28 °C.

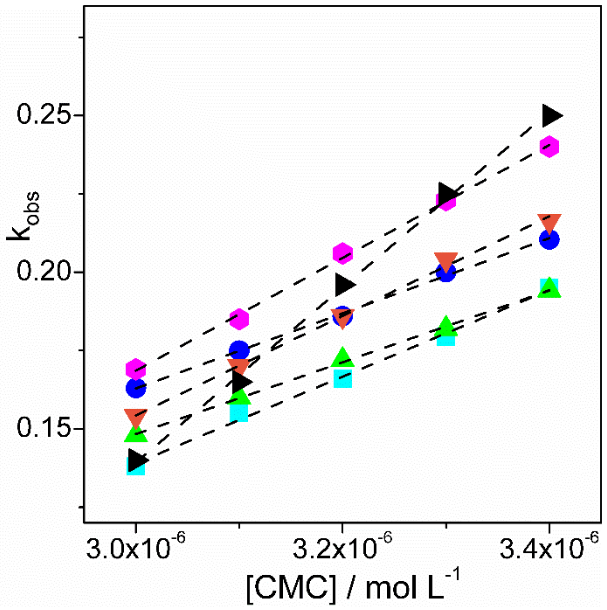

Figure S2. Plot of k_{obs} as a function of CMC concentration, used to determine k_a at the following temperatures: (■) 12 °C, (●) 16 °C, (▲) 20 °C, (▼) 24 °C, (⬢) 25 °C and (▶) 28 °C.

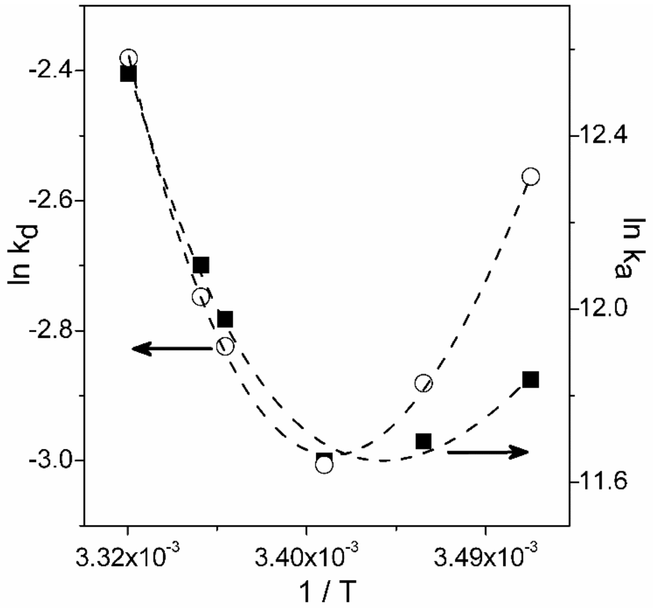

Figure S3. Arrhenius plots of ln k_a (■) and ln k_d (○) as a function of the reciprocal temperature (in Kelvin), associated with the BLF-CMC activated complex at pH 4.0.

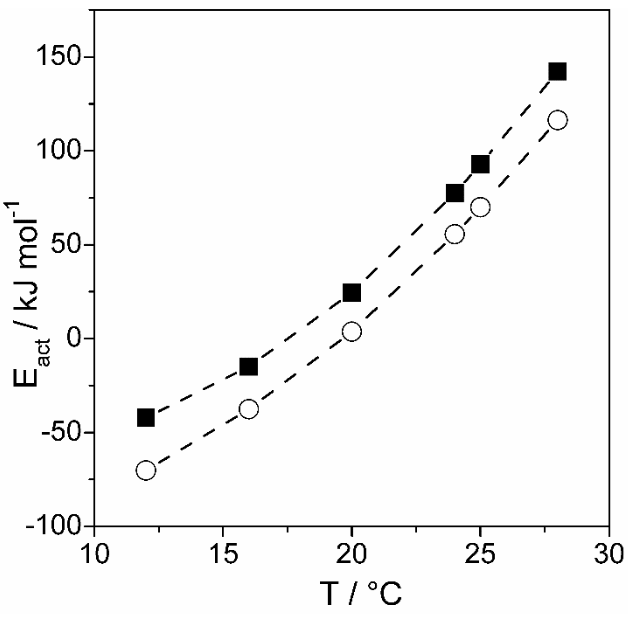

Figure S4. Activation energy *versus* temperature for the formation of the activated complex from the: (■) association of free BLF and CMC biomolecules and (○) dissociation of the BLF-CMC stable complex.

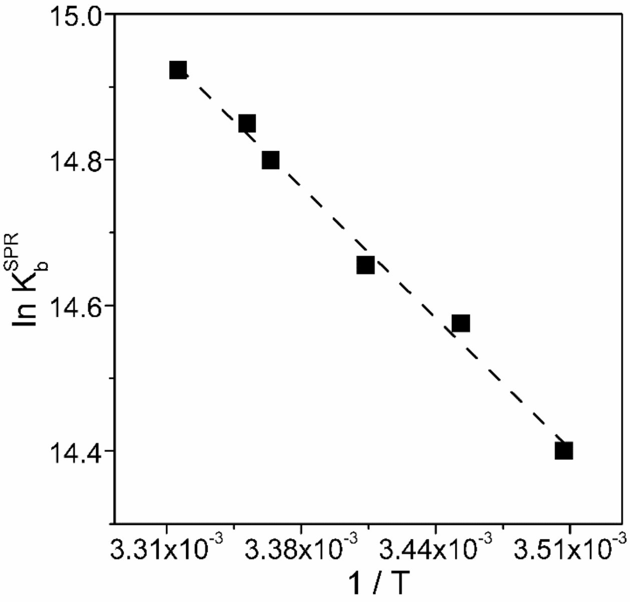

Figure S5. van't Hoff plot (ln K_b^{SPR} as a function of $1/T$ (K)) for the interaction between BLF and CMC studied by SPR at pH 4.0.

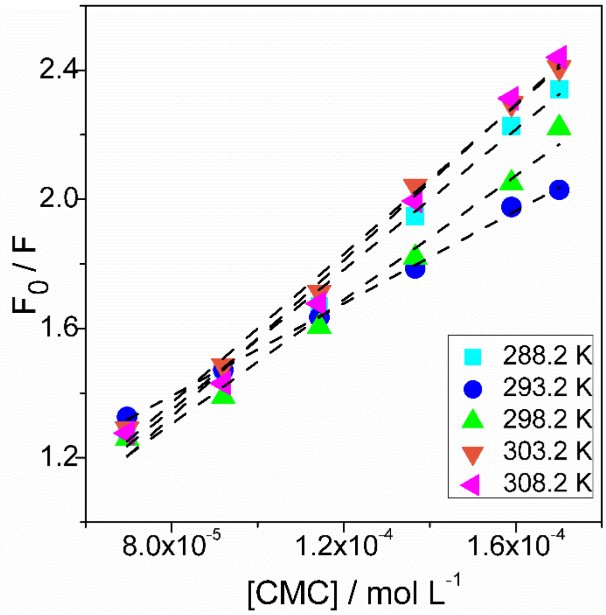

Figure S6. Stern-Volmer plots for BLF fluorescence quenching induced by binding to CMC, at pH 4.0.

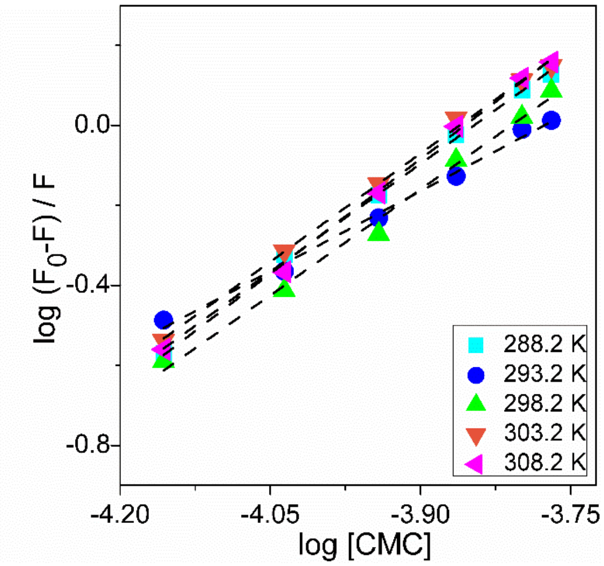

Figure S7. Plot for the determination of K_b and n values for the BLF-CMC complex at pH 4.0.

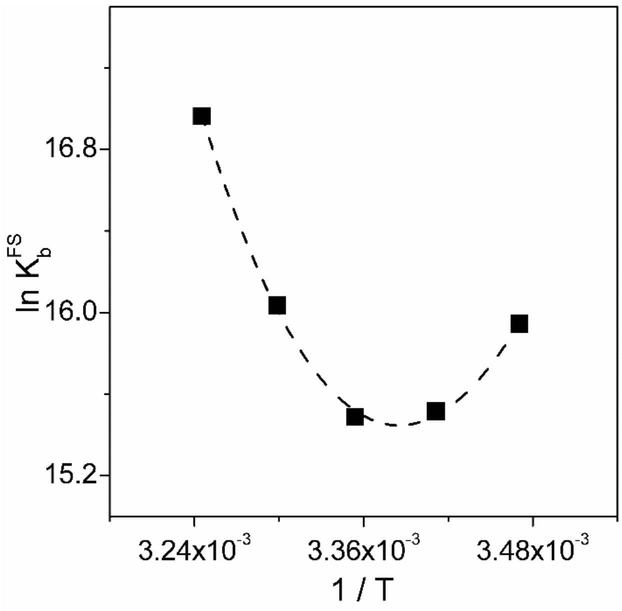

Figure S8. van't Hoff plot (ln K_b^{SPR} versus $1/T$ (K)) for the interaction between BLF and CMC studied by FS at pH 4.0.

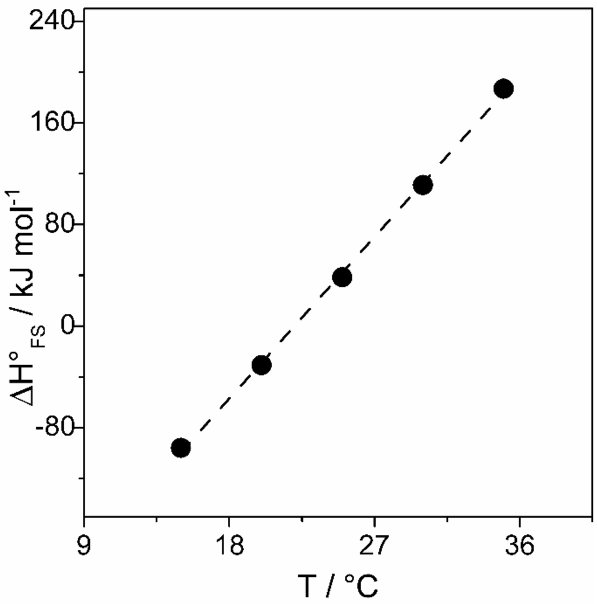

Figure S9. ΔH_F^o *versus* T plot for the BLF-CMC interaction obtained by fluorescence spectroscopy.

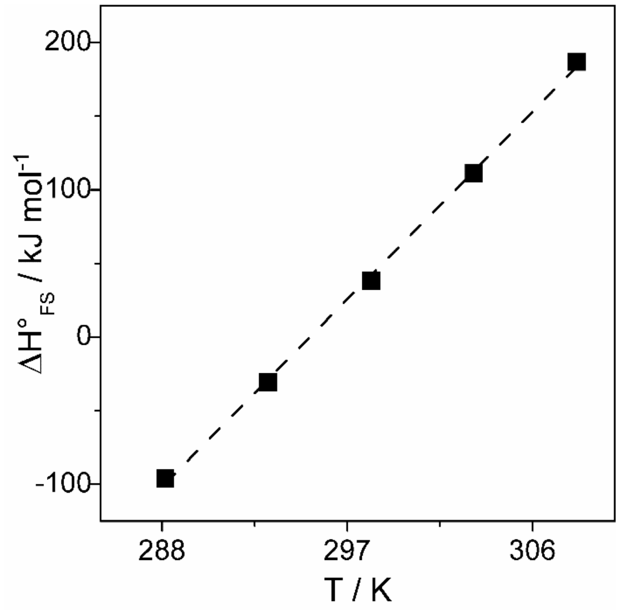

Figure S10. ΔH_F^o *versus* T (K) plot for BLF-CMC interaction obtained by fluorescence spectroscopy.

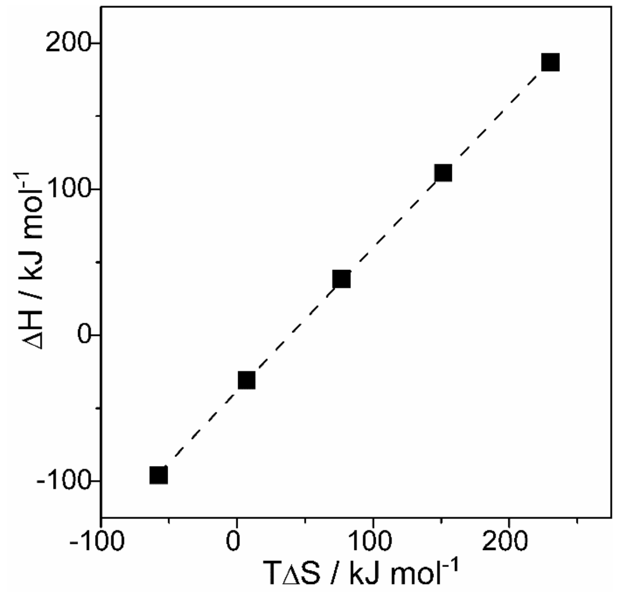

Figure S11. Enthalpy-entropy compensation for the BLF-CMC binding process examined by the fluorescence technique.

Table S1. Rate constants for the association (k_a) between free BLF and CMC biomolecules and the dissociation (k_d) of the BLF-CMC stable complex at pH 4.0 and different temperatures.

T	k_a	k_d
°C	10^5 L mol^{-1} s^{-1}	10^{-2} s^{-1}
12	1.4 ± 0.1	7.7 ± 0.1
16	1.2 ± 0.1	5.6 ± 0.1
20	1.2 ± 0.1	4.9 ± 0.2
24	1.6 ± 0.1	5.9 ± 0.2
25	1.8 ± 0.1	6.4 ± 0.1
28	2.8 ± 0.1	9.3 ± 0.3

Table S2. Bimolecular quenching rate constant (k_q), binding constants (K_b^{FS}), and stoichiometry numbers (n) for complex formation between BLF and CMC at different temperatures and pH 4.0.

T	k_q	K_b^{FS}	n	r^2
°C	10^{12} L mol^{-1}s^{-1}	10^6 L mol^{-1}		
15	10.3 ± 0.4	8.40 ± 0.01	1.8 ±0.1	0.997
20	6.8 ± 0.1	5.47 ± 0.02	1.4 ± 0.1	0.992
25	9.1 ± 0.5	5.32 ± 0.02	1.8 ± 0.1	0.991
30	10.9 ± 0.4	9.19 ± 0.01	1.8 ± 0.1	0.997
35	11.4 ± 0.6	23.28 ± 0.01	2.0 ± 0.1	0.997
